# School violence, depression symptoms, and school climate: a cross-sectional study of Congolese and Burundian refugee children

**DOI:** 10.1186/s13031-022-00475-9

**Published:** 2022-07-23

**Authors:** Camilla Fabbri, Timothy Powell-Jackson, Baptiste Leurent, Katherine Rodrigues, Elizabeth Shayo, Vivien Barongo, Karen M. Devries

**Affiliations:** 1 London School of Hygiene and Tropical Medicine, 15-17 Tavistock Pl, London, WC1H 9SH UK; 2 International Rescue Committee, 122 E 42nd St, New York, NY 10168 USA; 3 National Institute for Medical Research, 3 Barack Obama Dr, P.O. Box 9653, 11101 Dar es Salaam, Tanzania; 4 University College London, London, UK

**Keywords:** Refugees, Violence, Depression, School climate, Children

## Abstract

Forcibly displaced children are at increased risk of violence and mental health disorders. In refugee contexts, schools are generally perceived as protective environments where children can build a sense of belonging and recover from trauma. Evidence shows that positive school climates can support student skills development and socio-emotional wellbeing and protect them against a host of adverse outcomes. However, schools are also places where children may experience violence, from both teachers and peers. Prevalence estimates of violence against children in humanitarian settings are scarce and evidence on the relationship between school climate and student outcomes in these contexts is non-existent. The aim of the study is to estimate the prevalence of school-based violence against children and to explore the association between perceptions of school climate and students’ experiences and use of violence and their depression symptoms. We relied on data from a cross-sectional survey of students and teachers in all primary and secondary schools in Nyarugusu Refugee Camp in Tanzania, conducted as part of a cluster randomised controlled trial, to compute prevalence estimates and used mixed logistic regression analysis to assess the association between school climate and students’ outcomes. We found that students in Nyarugusu experienced high levels of violence from both peers and teachers in both primary and secondary schools in the camp, with little difference between boys and girls. Nearly one in ten students screened positive for symptoms of depression. We found that opportunities for students and teachers to be involved in decision-making were associated with higher odds of violent discipline and teachers’ self-efficacy was a significant protective factor against student depression symptoms. However, generally, school-level perceptions of school climate were not associated with student outcomes after adjusting for potential confounders. Our findings suggest that interventions to prevent and respond to teacher and peer violence in schools and to support students’ mental health are urgently needed. Our results challenge the assumption that education environments are inherently protective for children and call for further investigation of norms around violence among students and teachers to better understand the role of school climate in refugee settings.

**Supplementary Information:**

The online version contains supplementary material available at 10.1186/s13031-022-00475-9.

## Introduction

Over half of the world refugees are children, and in Africa alone there are 4 million child refugees [1], who are often separated from their family members and have suffered violence and abuse. Tanzania hosts nearly than 250,000 refugees from the Democratic Republic of Congo (DRC) and Burundi across three camps located in the north-western region of Kigoma. More than half of the refugees in Tanzania are children whose experiences of violence, economic insecurity, and forced displacement expose them to compounded risks for their physical, emotional, and social development [2, 3].

For refugee children, school can play a crucial protective and therapeutic role offering an opportunity to learn, play and build relationships while recovering from trauma [4–6]. School environments that promote physical and emotional safety, respectful relationships, supportive teaching and learning practices, and that allow individuals to contribute to a shared vision and common rules within the school are associated with a host of positive outcomes for students [7, 8]. Positive school climates can influence student academic outcomes [9–12] and rates of violence [13–16], support skills development [17, 18] and reduce risk of mental health disorders [19–21]. Conversely, negative educational environments can be predictive of students’ depression symptoms [22], low self-esteem [23] and indicators of alienation from school are related to substance abuse behaviours [24].

Positive school environments are particularly important for children at increased risk of feeling alienated or isolated [25] such as displaced children. School offers opportunities for socialisation and promotes a sense of belonging, which may be undermined by forced displacement and separation from family and communities. A greater attachment and increased social connection to school are protective against internalising problems and depression [26, 27]. Building supportive relationships in school further contributes to promoting values for peacebuilding that enable displaced children to develop resilience and coping mechanisms [28]. However, school is also where children may experience violence, and teachers and peers are common perpetrators across a range of countries [29].

Representative prevalence surveys from East Africa show that violence in and around schools is widespread and both peers and teachers were identified as common perpetrators of violence [30]. In refugee settings, children may be at increased risk of violence due to overcrowding and resource constraints, a general lack of security, political tensions, and weak child protection referral and response systems [31, 32]. In these contexts, school violence may be further exacerbated by teachers’ high levels of stress and histories of trauma, which may affect their use of violent discipline and ability to effectively supervise students in class [33, 34]. Norms that promote or condone use of violence may also contribute to high levels of teacher and peer violence [35].

Despite increasing research on the forms of violence that children experience in humanitarian settings, official prevalence estimates do not exist and the relationship between school climate, violence, and children’s outcomes in these contexts remains largely unexplored [36]. A recent review of risk and protective factors of refugee children’s mental health identified only one study that included school and peer-relationships [2]. Although it’s widely accepted that schools play a protective role in settings characterised by violence and under-provision of health and support services for children, available evidence on rates of school violence and children’s feelings of safety in school complicates this assumption and warrants further investigation of the role that school environments play in the lives of refugee children [37]. Moreover, evidence on how dimensions of school climate identified in research conducted in the UK and the US translate to low- and middle-income settings is scant and generally of poor quality [38], and tools used to assess dimensions of school climate are often adapted from high income contexts with limited consideration of how local cultural and social norms influence what is perceived as valuable in the school environment [39].

In this study we used survey data collected from students and teachers at baseline, as part of a cluster randomised trial evaluating the effectiveness of a school-based violence prevention intervention in Nyarugusu Refugee Camp in Tanzania [40], to estimate the prevalence of school violence and depression symptoms among school children and adolescents and we explored the association between student outcomes and dimensions of school climate, based on the hypothesis that positive school climates would be associated with students’ feelings of safety in school and reduced misbehaviours, which could lead to lower levels of violence and depression. We hypothesised that school environments that promote caring relationships between students and teachers, characterised by high levels of trust, that offer opportunities to engage in decision making and to develop skills, would make both students and teachers feel more connected and empowered reducing opportunities for conflict and for experiencing adverse mental health outcomes.

## Methods

### Study setting

The study took place in Nyarugusu Refugee Camp in Kigoma, Tanzania, which was established in 1996 to host refugee populations fleeing conflict in the Democratic Republic of Congo. According to the latest UNHCR data, the camp hosts nearly 130,000 refugees [41], making it one of the largest in the world. The camp is largely divided by country of origin, and schools specifically serve Congolese or Burundian populations following two different curricula. School enrolment is higher than in other refugee settings, with nearly 78% of students enrolled in school [42].  Although 70% of students reported feeling safe in school; several child protection risks were  identified in and around schools [42]. The quality of service provision is further affected by situations of overcrowding and resource constraints and existing qualitative data additionally revealed that violent discipline is prevalent in Nyarugusu schools and that teachers perceive that hitting students can help them learn how to behave [43].

### Participants

We used data from the baseline survey of the Preventing Violence Against Children in Schools (PVACS) study which is a cluster randomized controlled trial assessing the effectiveness of the EmpaTeach intervention to change teacher practices and reduce use of violence in schools [40]. A cross-sectional survey was conducted between November and December 2019 with a representative sample of teachers and students in all 27 primary and secondary schools in the camp. Consent for data collection and to approach individual students was sought from the headteacher in each school. Students provided informed assent, and individual teachers provided informed consent. In each school, 60 students and 20 teachers were randomly selected from up-to-date lists of all teachers and students in the school; the sample for students was stratified by age to ensure representation of all age groups. Surveys were conducted with students in grade 2 or above and aged 9 years and over, as they were able to understand and respond to questions during pre-testing of the instruments. Additional details on sampling and consent procedures are described elsewhere [40].

All interviews were administered by trained enumerators using tablet computers. Children were informed during the consent process that the research team would need to pass their details on to child protection officers if they disclosed experiences of violence. The student survey included algorithms that prompted different interview finishes to guide enumerators through the appropriate referral pathway based on the student age and responses to survey questions. Referral protocols were based on predefined criteria (such as time frame and type of abuse) agreed on with IRC’s child protection team. All students were offered counselling regardless of what was disclosed during the survey. Teachers were offered referral to IRC services available within the camp.

Participants were not involved in setting up the PVACS trial but were involved in various phases of formative research conducted by the IRC that informed the design of the study. Refugee communities from Nyarugusu were involved in dissemination of the trial findings.

### Measures

#### Perceptions of school climate

Sets of items were selected from the teacher survey on the basis of frameworks proposed by Cohen [44] to capture perceptions of school climate across various domains of the school environment. We modelled these exposures as continuous variables and assessed their psychometric properties (results are presented in Additional file 1: Table S1). Table 1 describes items included under each of the school-climate measures used in the analyses: quality of relationships among members of the school community as reported by teachers, teachers’ job satisfaction and wellbeing at school, teachers’ self-efficacy, and teacher-reported opportunities for involvement in decision making for staff and students. All school-climate variables ranged from1 to 4 and for all indicators a higher score corresponded to a more positive school climate domain (e.g., a high relationship score reflecting more supportive relationships between teachers and students).

**Table 1:** Description of school climate measures included in analyses

Measure	Description and item wording (response options)
Teacher-student relationships *Adapted from the Good School Study* [45]	“Do you feel that students respect their peers and adults?”, “Do you feel that school staff respect their students?”, “Do you have a good relationship with the parents?”, “Do you have a good relationship with the students?”, “Would you say that students feel comfortable talking to you/want to confide in you if they are unhappy about something at home or at school?”, “Thinking about your school as a whole, do you feel like you are part of a team?”. (“all the time”, “most of the time”, “sometimes”, “never”)
Teacher self-efficacy *Adapted from Teachers' Sense of Efficacy Scale* [46]	“Do you feel capable to help students believe they can do well in school work?”, “Do you feel capable to help your students value learning?”, “Do you feel capable to write good questions for your students?”, “Do you feel capable to control disruptive behaviour in the classroom?”, “Do you feel capable to motivate students who show low interest in school work?”, “Do you feel capable to make your expectations about how students should behave clear?”, “Do you feel capable to help your students think critically?”, “Do you feel capable to get your students to follow classroom rules?”, “Do you feel capable to calm a student who is disruptive or noisy?”, “Do you feel capable to use a variety of strategies to evaluate your students?”, “Do you feel capable to provide an alternative explanation or example when students are confused?”, “Do you feel capable to use different teaching methodologies?”. (“not at all capable”, “a little capable”, “capable”, “very capable”)
Teacher job satisfaction *Adapted from the Good School Study* [45]	“How often would you say you feel that you enjoy your job?”, “Do you feel adequately rewarded financially for what you do?”, “Do you feel valued as an employee?”, “Do you take pride in your work?”, “Do you feel that your employers care about your wellbeing?”. (“all the time”, “most of the time”, “sometimes”, “never”)
Teacher and student involvement in school operations *Adapted from the Good School Study* [45]	“Do you have enough opportunities to say what you think and contribute to how the school is run?”, “Do you feel that your views on how the school's policies could be improved are welcomed?”, “How often do you take any actions to change how your school is run?”, “Do students in your school have an opportunity to say what they think”, “Do students in your school have an opportunity to contribute to how the school is run?”. (“all the time”, “most of the time”, “sometimes”, “never”)

#### Individual factors

We controlled for student characteristics that were considered possible confounders, including their socio-demographic characteristics such as sex, age modelled as a continuous variable, number of meals per day, disability status in the form of any functional difficulty reported [47], number of adults and children they shared sleeping areas with, whether they lived with at least one biological parent and family connectedness.

#### Outcomes

Outcome variables were selected to capture students’ experiences of school violence and depression symptoms. Students’ self-reports of violence from teachers and peers in the past week were measured using items adapted from the International Society for the Prevention of Child Abuse and Neglect Child Abuse Screening Tool-Child Institutional [48]. Questions on experiences of specific acts of violence were used to build two binary variables indicating whether students had experienced any emotional and/or physical violence from teachers in the past weeks, and any emotional, physical and/or sexual violence from peers in the past week. An indicator of students’ own aggressive behaviours towards peers was defined as a binary variable taking the value of one if the student reported any perpetration of violence towards peers in the past week. The outcome was based on students’ self-reported use of violence and individual students were categorised as having perpetrated violence against peers if they reported at least one of three acts of violence in the past week: (1) having used physical violence like hitting or slapping a peer, (2) having hit a peer with an object, chocked or burnt them or used a weapon against them, (3) having threatened or forced a peer to have sex. Finally, the short version of the Mood and Feelings Questionnaire Child Self-Report (SMFQ) [49] was used to measure students’ depression symptoms. The instrument showed good reliability in our sample with a Cronbach’s score of 0.84 and while there is no standardised cut-off for the SMFQ, in line with previous research we used scores above equal or above 12 to generate a binary variable indicating positive screening for symptoms of depression [50, 51]. Students’ experience of violence from peers and own perpetration of violence outcomes were available only for children aged 10 years and above. Additional file 1: Table S2 shows the items used to build each outcome variable.

### Statistical analysis

We first report descriptive statistics of school, teacher, and student characteristics including all school-level measures of school climate. We estimated prevalence of school violence and depression symptoms among students, disaggregated by sex and school level. Students' self-reported experiences of emotional, physical, and sexual violence perpetrated by teachers and peers were calculated as the proportion of students reporting lifetime and past week experience of at least one act of violence. Prevalence of student aggression was measured as the proportion of students who reported using at least one form of violence against peers in the past week. Mental health estimates relied on the proportion of students who screened positive for symptoms of depression.

We calculated the crude and adjusted associations between each of the school-level dimensions of school climate and each of the four outcomes using mixed logistic regression models. Adjusted models included student background characteristics as covariates (student sex, age, meals eaten yesterday, disability status, number of adults in same sleeping area, number of children in same sleeping area, living with at least one biological parent, family connectedness) as well as level of schooling and school nationality. The school-level measures of school climate were created by averaging teacher responses in each school. We then estimated the associations between perceptions of school climate and students' outcomes in a set of further adjusted mixed models (one for each outcome) that included all school-level variables and individual level confounders. All models included a random effect for schools to account for the clustered nature of the data (students were nested within schools). All analyses were conducted in Stata SE17 and were adjusted for stratified sampling.

## Results

### Descriptive statistics

As shown in Table 2 there were 27 schools in the camp, 21 primary and 6 secondary. On average, schools were characterised by high scores for teacher self-efficacy and quality of student and teacher relationships (mean score above 3) and lower scores for teacher job satisfaction and opportunities for involvement. Within schools, girls made up for 47% of the student population and the mean age was 13 years. Food security among students was low, with 16% of them having had no or one meal only on the previous day. The average child reported sharing a sleeping area with 3 adults and more than 4 children. 75% of students reported living with at least one biological parent and 42% reported having some form of functional disability. School attendance was higher among Congolese students, due to the fact that Congolese schools are open 6 days a week while the Burundian school week is only 5 days.

**Table 2:** School, teacher and student characteristics

	Freq (%), Mean (SD)	
Schools (N = 27)		
Level		
Primary	21	78%
Secondary	6	22%
School nationality		
Congolese	17	63%
Burundian	10	37%
Perceptions of school climate		
Teacher-student relationship	3.23	(0.05)
Teacher self-efficacy	3.26	(0.04)
Teacher job satisfaction	2.83	(0.75)
Teacher and student involvement in school operations	2.69	(0.06)
Teachers (N = 488)		
Sex		
Male	355	73%
Female	133	27%
Age group		
Below 30	214	44%
30–39	153	31%
40–49	52	11%
50 and above	69	14%
Country of origin		
DRC	303	62%
Burundi	184	38%
Meals eaten yesterday		
No meals	9	2%
One meal	164	33%
Two meals or more	315	65%
Students (N = 1493)		
Sex		
Male	799	53%
Female	694	47%
Age group		
10 or below	453	28%
11 to 14	643	45%
15 to 20	341	26%
21 or above	56	1%
Country of origin		
DRC	927	66%
Burundi	565	34%
Meals eaten yesterday		
No meals	8	1%
One meal	246	15%
Two meals or more	1239	84%
Number of adults in same sleeping area	3.05	(1.62)
Number of children in same sleeping area	4.65	(2.16)
Lives with at least one biological parent	341	75%
Has functional difficulty	654	42%
School attendance in past week		
DRC (of 6 days)	4.53	(0.91)
Burundi (of 5 days)	4.99	(1.55)

All estimates are adjusted for sampling weights

### Prevalence of school violence and depression symptoms among students

Students in Nyarugusu Refugee Camp were exposed to high levels of violence from both teachers and peers (Table 3). More than 80% of students had lifetime experiences of physical violence from school staff, with 84% of boys and 77% of girls reporting exposure. More than 55% of students had experienced physical violence from teachers in the past week, with the prevalence reaching nearly 60% in primary schools. Emotional violence also appeared frequent in Nyarugusu schools with 18% of students overall reporting past week exposure. Around 15% of students experienced physical or emotional violence from peers in the past week; and episodes of peer victimisation, both in the form of physical and emotional violence, generally affected boys more than girls. Around 4% of students experienced some form of sexual violence from peers in the past week. One in three students reported aggressive behaviours in the past week against peer students, with similar proportions across boys and girls. Perpetration of peer violence was more frequently reported by students in primary school (36%) compared to students in secondary school (31%). Overall, nearly one in ten students screened positive for depression symptoms. Symptoms of depression appeared to affect boys and girls similarly.

**Table 3:** Prevalence of school violence and student depression

	Freq (%)									
	Total		Male		Female		Primary		Secondary	
School violence										
Violence from school staff										
Emotional violence, lifetime	512/1493	34%	292/799	36%	220/694	31%	376/1166	32%	136/327	39%
Emotional violence, past week	275/1493	18%	144/799	17%	131/694	19%	218/1166	18%	57/327	17%
Physical violence, lifetime	1169/1493	81%	649/799	84%	520/694	77%	953/1166	82%	216/327	78%
Physical violence, past week	807/1493	56%	436/799	57%	371/694	55%	693/1166	59%	114/327	47%
Sexual violence, lifetime	30/1040	3%	15/574	3%	15/466	4%	15/713	3%	15/327	5%
Outcome: Any violence from school staff, past week	868/1493	60%	472/799	61%	396/694	58%	729/1166	62%	139/327	53%
Violence from peers										
Emotional violence, lifetime	309/1039	31%	188/574	35%	121/465	26%	211/713	30%	98/326	31%
Emotional violence, past week	151/1039	15%	82/574	15%	69/465	15%	113/713	17%	38/326	12%
Physical violence, lifetime	268/1040	28%	186/574	35%	82/466	19%	205/713	29%	63/327	25%
Physical violence, past week	142/1040	14%	92/574	17%	50/466	11%	119/713	17%	23/327	9%
Sexual violence, lifetime	88/1040	8%	46/574	9%	42/466	7%	47/713	8%	41/327	8%
Sexual violence, past week	39/1040	4%	18/574	4%	21/466	4%	24/713	4%	15/327	3%
Outcome: Any violence from peers, past week	243/1040	24%	141/574	26%	102/466	22%	188/713	27%	55/327	18%
Outcome: Aggressive behaviours in past week	357/1040	34%	201/574	36%	156/466	33%	264/713	36%	93/327	31%
Mental health										
Outcome: Depression symptoms, past week	132/1493	8%	72/799	8%	60/694	8%	88/1166	8%	44/327	10%

Questions about sexual violence from teachers, any violence from peers and students' own use of aggressive behaviours were not asked to children under 10 years of age (N = 1040)

### Associations between perceptions of school climate and student outcomes

Table 4 reports the results of the crude and adjusted associations between each of the school climate domains and each of the outcomes. In the unadjusted analysis, positive perceptions of school climate as measured by quality of teacher-student relationships, teacher self-efficacy, teacher job satisfaction, and opportunities for participation in decision making were generally significantly associated with students’ experiences of violence from teachers and depression symptoms, but not with peer victimisation and students’ own perpetration of violence (teacher job satisfaction was significantly associated only with teacher violence). Positive relationships between teachers and students, higher job satisfaction among teachers, and more opportunities for involvement in decision making for students and teachers were all associated with higher odds of violence from teachers, while higher levels of self-efficacy reported by teachers were associated with lower odds of teacher violence. Better school-level scores for quality of relationships, job satisfaction, and involvement in decision making were also significantly associated with lower odds of symptoms of depression in students. After adjusting for individual student covariates, the only statistically significant domains remained the school-level involvement of teachers and students in school operations and the level of teacher self-efficacy. The odds of experiencing any violence from teachers was more than double as high with each unit increase in the opportunities for involvement score (OR 2.54, 95% CI 0.90–7.18) and each increase in the score for teacher self-efficacy was associated with a 94% decrease in the odds of depression symptoms for students (OR 0.06, 95% CI 0.01–0.27).

**Table 4:** Crude and adjusted associations between each school-level measure of school climate and students' outcomes

School-level exposures	Outcomes															
	Any violence from teachers				Any violence from peers				Aggressive behaviours				Depression symptoms			
	Unadjusted		Adjusted		Unadjusted		Adjusted		Unadjusted		Adjusted		Unadjusted		Adjusted	
	OR (95% CI)	*P*	OR (95% CI)	*P*	OR (95% CI)	*P*	OR (95% CI)	*P*	OR (95% CI)	*P*	OR (95% CI)	*P*	OR (95% CI)	*P*	OR (95% CI)	*P*
Teacher-student relationship	4.10 (1.75 to 9.60)	0.002	0.86 (0.35 to 2.11)	0.738	1.34 (0.56 to 3.20)	0.503	1.33 (0.43 to 4.06)	0.606	0.78 (0.28 to 2.13)	0.614	0.44 (0.10 to 1.93)	0.267	0.30 (0.13 to 0.72)	0.009	2.15 (0.41 to 11.31)	0.349
Teacher self-efficacy	0.25 (0.07 to 0.94)	0.041	0.57 (0.29 to 1.67)	0.295	1.57 (0.57 to 4.32)	0.367	1.15 (0.30 to 4.38)	0.833	1.13 (0.32 to 4.00)	0.842	0.78 (0.13 to 4.67)	0.783	1.99 (0.42 to 9.49)	0.373	0.06 (0.01 to 0.27)	0.001
Teacher job satisfaction	2.26 (1.30 to 3.92)	0.006	1.04 (0.53 to 2.06)	0.897	1.09 (0.59 to 2.01)	0.767	1.22 (0.60 to 2.48)	0.564	0.77 (0.38 to 1.58)	0.463	0.60 (0.22 to 1.64)	0.310	0.44 (0.17 to 1.11)	0.079	1.28 (0.41 to 4.01)	0.654
Teacher and student involvement in school operations	3.65 (1.77 to 7.50)	0.001	2.54 (0.90 to 7.18)	0.078	0.91 (0.46 to 1.78)	0.766	0.63 (− 0.44 to 1.69)	0.238	0.95 (0.44 to 2.09)	0.901	1.47 (0.33 to 6.53)	0.602	0.29 (0.10 to 0.84)	0.024	1.31 (0.26 to 6.47)	0.727

N for unadjusted analyses = 1495. N for adjusted analyses = 1484. Adjusted analyses include student sex, age, meals eaten yesterday, disability status, number of adults in same sleeping area, number of children in same sleeping area, living with at least one biological parent, home connectedness, school level and school nationality

In the full models that included all domains of school climate, most of the school-level variables capturing perceptions of school climate were not significantly associated with students’ experiences of violence in school and depression symptoms (Table 5). Involvement of students and teachers in decision-making remained significantly associated with higher levels of violence from teachers (OR 4.24, 95% CI 1.10–16.33) and schools where teachers were more confident of their skills had significantly lower odds of depression symptoms among students (OR 0.04, 95% CI 0.01–0.24). The school climate domains were not significantly associated with peer victimisation or with students’ own perpetration of violence. The quality of relationships between teachers and students and teachers’ job satisfaction were found to be not associated with students' outcomes. Being in a secondary school was a protective factor against experiences of school violence from peers (OR 0.50, 95% CI 0.26–0.99), and children in Congolese schools appeared to have lowers odds of experiencing depression symptoms (OR 0.20, 95% CI 0.06–0.61). Student covariates were generally not associated with student outcomes (results not shown) with few exceptions: coming from crowded households was positively associated with students’ own aggressive behaviours (OR 1.08, 95% CI 1.00–1.17), and reporting good home connectedness was associated with lower odds of experiencing violence from peers (OR 0.56, 95% CI 0.38–0.83) and of screening positive for depression symptoms (OR 0.53, 95% CI 0.31–0.88).

**Table 5:** Full models of associations between perceptions of school climate and students' outcomes

School-level exposures	Any violence from teachers	Any violence from peers	Aggressive behaviours	Depression symptoms
	OR (95% CI)	OR (95% CI)	OR (95% CI)	OR (95% CI)
Teacher-student relationship	0.55 (0.16–1.93)	0.98 (0.23–4.11)	0.41 (0.07–2.52)	4.47 (0.37–53.43)
Teacher self-efficacy	0.52 (0.18–1.45)	1.18 (0.26–5.32)	0.63 (0.11–3.61)	0.04*** (0.01–0.24)
Teacher job satisfaction	0.80 (0.37–1.73)	1.13 (0.45–2.85)	0.53 (0.17–1.64)	0.57 (0.14–2.38)
Teacher and student involvement in school operations	4.24** (1.10–16.33)	1.43 (0.19–10.57)	3.92* (0.86–17.90)	1.14 (0.19–6.99)

N for violence from teachers and depressive symptoms = 1493. N for violence from peers and aggressive behaviours = 1040. All models include student sex, age, meals eaten yesterday, disability status, number of adults in same sleeping area, number of children in same sleeping area, living with at least one biological parent, home connectedness, school level and country of origin. ***p < 0.01; **p < 0.05; *p < 0.1

## Discussion

In this paper we presented first-time prevalence estimates of school violence and depression symptoms among a representative sample of students from one of the largest refugee camps globally and examined their associations to various dimensions of school climate. First, we showed that refugee children in Nyarugusu Refugee Camp were exposed to high levels of violence from both teachers and peers. Nearly one in two students experienced physical violence from teachers in the past week and one in four experienced peer victimisation. Acts of emotional violence affected between 15 and 35% of children in the past week and both teachers and peers appeared as common perpetrators. Students were also at risk of sexual abuse with between 3 and 8% of students reporting some experience of sexual violence from teachers and/or peers. While the emotional and physical violence estimates were in line with prevalence rates from other settings in Sub-Saharan Africa, rates of sexual violence in childhood and in the past week were higher than in other settings [52] and similar between boys and girls. We found that nearly one in ten students screened positive for depression symptoms which could be linked to a variety of child protection risks children were exposed to in the camp as well as to past experiences of trauma. These estimates were consistent with the WHO age-standardised prevalence rates of depression for displaced and conflict-affected populations [53]. The evidence on the negative effects of adverse experiences in childhood is well established and exposure to violence, as well as to stressors related to displacement and the refugee experience, are known risk factors for developing mental health disorders [54, 55]. To date only one intervention to prevent and reduce school violence in humanitarian settings has been rigorously tested [40] and while the evidence on effective school-based ways to support mental health and psychosocial wellbeing of refugee children is growing, there remain large evidence gaps [56, 57].

We hypothesised that a positive school climate would act as a protective factor against these adverse school experiences; we postulated that perceptions of school climate, as measured from teachers, would be associated with lower levels of school violence and depression among students. We found that our results did not substantiate these hypotheses. School-level factors were generally not associated with students’ outcomes with the exception of inclusive decision making and teachers’ self-efficacy. Opportunities for engagement for students and teachers were associated with higher levels of school violence and higher scores of teachers’ self-efficacy were associated with lower odds of depression symptoms for students. While the first finding may appear surprising, in contexts where corporal punishment is not only condoned but is perceived as an effective and acceptable form of discipline, opportunities to contribute to decision making in the school for students and teachers may result in higher levels of violence [58]. On the contrary, in schools where involvement of refugees in decision making is limited and where policies are made directly by school administrators in compliance with camp regulations use of violence may be less common. The association between teaching ability and student depression echoes findings from previous studies where school-level measures of teacher wellbeing were associated with lower rates of depression among students [59] suggesting that schools with more structured disciplinary styles and where teachers feel more confident about their own skills and ability to manage students may be able to better support students’ emotional wellbeing [18].

Overall, our results are consistent with evidence from non-crisis settings; although there is ample empirical evidence on the association between individual-level perceptions of school climate and students’ experiences of violence and mental health disorders, several studies found no or little association with school-level measures of climate [59–61]. Whilst our study design did not allow us to explore the relationship between students’ own perceptions of school climate and their social and health outcomes, our findings offer some initial insights into the role of school environment in refugee settings.

Importantly, our results can inform the development of effective school-based interventions to prevent violence and improve student wellbeing in humanitarian settings. In Nyarugusu, students in Congolese schools appeared to be significantly less likely to suffer from depression symptoms and this may be due to the fact that Congolese refugees have been in the camp for decades and may have a more settled and stable life in the camp compared to Burundian students, whose experiences of conflict and displacement may be more recent. Generally, students had lower odds of experiencing and perpetrating violence in secondary schools, suggesting that violence and bullying prevention may be more urgent among younger children. Taken together these results underscore the need for effective school-based interventions targeted at displaced and refugee children and adolescents, whose adverse experiences in school may compound existing trauma leading to negative long term outcomes [62].

Our study had several strengths and some limitations. We used data from a representative sample of students and teachers from one of the largest refugee settings globally, therefore our findings are generalisable to the general camp population of teachers and students. Although survey tools used in the study were not originally created to assess quality of school climate, we were able to operationalise most of the widely recognised dimensions of school climate. We assessed measures for consistency at the school level and constructed reliable indicators of school climate. Our psychometric assessment led to inclusion of school-level indicators that were built solely from teachers’ perceptions of school climate which were therefore exogenous to students' outcomes and reduced risk of unobserved heterogeneity bias. However, the lack of student-informed measures of school climate may be considered a limitation of our study given that, from a conceptual perspective, school climate represents the shared values, norms and experiences of the whole school community, which includes students as well. Additionally, our study relied on a cross-sectional design, therefore we cannot make causal inferences from the associations presented. Although students provided informed assent to participate in the study and they were informed about the confidential nature of their responses, stigma and concerns around confidentiality as well as social desirability bias may have resulted in under-reporting of sensitive outcomes such as experiences of violence and depression symptoms.

Our analyses have shown that while schools can act as an important protective environment for children, child refugees may also endure a host of adverse school experiences with negative consequences for their physical and mental health. The fact that individual student characteristics were generally not associated with their experiences and use of violence suggests that violence in schools in Nyarugusu Camp is normalised and not targeted at or used by specific groups. In this context, child protection and mental health interventions targeted at children and adolescents are urgently needed and schools offer an ideal service delivery platform to reach large numbers of children given the higher enrolment rates that characterise some protracted humanitarian crises [63].

## Supplementary Information

**Additional file 1.** Additional intomation on school-climate exposures and students' outcomes.

## Data Availability

Fully anonymised data are available on request from the LSHTM Data Repository (https://doi.org/10.17037/DATA.00002474) for researchers who meet the criteria for data access and whose intended analyses fall under the scope of the PVAC study. The LSHTM Research Data Manager, based in the Library & Archives Service, is responsible for managing data in the repository and can be contacted at researchdatamanagement@lshtm.ac.uk.
