# Supplementary material for: School violence, depression symptoms, and school climate: a cross-sectional study of Congolese and Burundian refugee children
Source: Confl Health. 2022 Jul 23;16:42. doi: 10.1186/s13031-022-00475-9 (PMC9308201; doi:10.1186/s13031-022-00475-9)
Supplement: Supplementary file 1 — Additional file 1. Additional intomation on school-climate exposures and students' outcomes. [file 13031_2022_475_MOESM1_ESM.docx]

# Supplementary information to the manuscript titled: School violence, depression, and school climate: a cross-sectional study of Congolese and Burundian refugee children

**Table S1. Psychometric assessment of school climate measures**

| Measure | Source | N | Items | Range | Mean | SD | Cronbach’s alpha | ICC |
| --- | --- | --- | --- | --- | --- | --- | --- | --- |
| Teacher-student relationship | Teacher | 488 | 6 items | 1-4 | 3.25 | 0.47 | 0.58 | 0.19 |
| Teacher self-efficacy | Teacher | 488 | 12 items | 1-4 | 3.28 | 0.41 | 0.93 | 0.17 |
| Teacher job satisfaction | Teacher | 488 | 5 items | 1-4 | 2.82 | 0.73 | 0.68 | 0.17 |
| Teacher and student involvement in school operations | Teacher | 488 | 5 items | 1-4 | 2.68 | 0.59 | 0.66 | 0.17 |

Internal consistency of individual responses to these scales - i.e. to what extent items included in each scale measured the same construct - was examined using Cronbach’s alpha and level of agreement between participants’ mean scores in each school was assessed using the within school intraclass correlation coefficient (ICC). Individual responses to each scale had a Cronbach’s alpha close to or above the 0.60 threshold (1). School climate reflects shared norms, values and practices; therefore, it was deemed appropriate to construct school-level indicators for measures that showed clustering within schools. All measures had an ICC above 0.1 which was in line with previous work for health and education behaviours in students (2).

**Table S2. Outcome measures**

| Measure | Description |
| --- | --- |
| Experiences of violence from teachers in past week  *(International Society for the Prevention of Child Abuse and Neglect Child Abuse Screening Tool-Child Institutional)* | Outcome coded 1 if answered yes to any of the items; coded 0 if answered no to all 34 items.  Cursed or called you bad names? Shouted or yelled at you? Insulted you or said something mean to you? Humiliated or embarrassed you? Talked about your skin colour/gender/religion/tribe or health problems you have in a hurtful way? Kept you away from other children to make you feel bad or lonely? Humiliated you because you were unable to buy things? Stole or broke or ruined your belongings? Threatened you with bad marks that you didn’t deserve? Hurt you or caused pain to you? Slapped you with a hand on your face or head as punishment? Slapped you with a hand on your arm or hand? Twisted your ear as punishment? Twisted your arm as punishment? Pulled your hair as punishment? Hit you by throwing an object at you? Hit you with a closed fist? Hit you with a stick? Hit you with a cane? Kicked you with a foot? Knocked you on the head as punishment? Hit your fingers or hands with an object as punishment? Crushed your fingers or hands as punishment? Made you stand/kneel in a way that hurts to punish you? Made you stay outside for example in the heat or rain to punish you? Burnt you as punishment? Taken your food away from you as punishment? Forced you to do something that was dangerous? Choked you? Tied you up with a rope or belt at school? Tried to cut you purposefully with a sharp object? Severely beat you up? |
| Experiences of violence from peers in past week  *(International Society for the Prevention of Child Abuse and Neglect Child Abuse Screening Tool-Child Institutional)* | Outcome coded 1 if answered yes to any of the items; coded 0 if answered no to all 11 items.  Insulted you, or called you rude or hurtful names? Twisted your arm or any other body part, slapped you, pushed you or thrown something at you? Punched you, kicked you, or hit you with a closed fist? Hit you with an object, such as a stick or a cane, or whipped you? Cut you with a sharp object or burnt you? Disturbed or bothered you by making sexual comments about you? Kissed you, when you did not want them to? Touched your genitals or breasts when you did not want them to, or in a way that made you uncomfortable? Threaten or pressure you to make you do something sexual with them? Make you have sex with them, because they threatened or pressured you? Had sex with you, by physically forcing you? |
| Aggressive behaviours | Outcome coded 1 if answered yes about to any of the items; coded 0 if answered no to all 3 items.  Have you ever used physical violence against anyone, like hitting, slapping, punching or kicking? Have you ever hit anyone with an object, choked, burnt, or used a weapon to hurt them? Have you ever threatened someone to make them or forced anyone to have sex or do sexual things with you? |
| Depressive symptoms  *Mood and Feelings Questionnaire Child Self-Report (SMFQ)* | Each of the 13 items was scored according to the scaled: 0=not true, 1=sometimes true, 2=true. Higher values indicated higher severity of depressive symptoms. Outcome coded 1 if summary score =>12.  In the past two weeks: you felt very sad; you didn’t enjoy anything at all; you could not do your normal activities because you felt so sad; you could not relax your mind; you felt worthless; you cried a lot; you found it hard to pay attention; you hated yourself; you were a bad person; you felt sad because you were alone with your thoughts; you thought nobody really loved you; you thought you could never be as good as other kids; you did everything wrong. |

**References**

1. Hulin C, Netemeyer R, Cudeck R. Can a reliability coefficient be too high? Journal of Consumer Psychology. 2001:55-8.

2. Shackleton N, Hale D, Bonell C, Viner RM. Intraclass correlation values for adolescent health outcomes in secondary schools in 21 European countries. SSM Popul Health. 2016;2:217-25.
